# Hybrid Deep Learning-Based Enhanced Occlusion Segmentation in PICU Patient Monitoring

**DOI:** 10.1109/OJEMB.2024.3503499

**Published:** 2024-11-20

**Authors:** Mario Francisco Munoz, Hoang Vu Huy, Thanh-Dung Le, Philippe Jouvet, Rita Noumeir

**Affiliations:** Electrical Engineering DepartmentÉcole de Technologie Supérieure 514734 Montréal QC H3C 1K3 Canada; Saint-Justine Mother and Child University Hospital Center Montréal QC H3C 1K3 Canada; Biomedical Information Processing LabÉcole de Technologie Supérieure, University of Québec 14849 Montréal H3C 1K3 Canada; Biomedical Information Processing LabÉcole de Technologie Supérieure, University of Québec 14849 Montréal H3C 1K3 Canada; Interdisciplinary Centre for Security, Reliability, and Trust (SnT)University of Luxembourg 81872 L-1855 Luxembourg; ^6^ CHU Sainte-Justine HospitalUniversity of Montreal 5622 Montréal H3C 1K3 Canada; Biomedical Information Processing LabÉcole de Technologie Supérieure, University of Québec 81872 Montréal H3C 1K3 Canada

**Keywords:** Computer vision, data augmentation, deep learning, model fusion, occlusions, pediatrics intensive care, remote patient monitoring (RPM), segmentation

## Abstract

Remote patient monitoring has emerged as a prominent non-invasive method, using digital technologies and computer vision (CV) to replace traditional invasive monitoring. While neonatal and pediatric departments embrace this approach, Pediatric Intensive Care Units (PICUs) face the challenge of occlusions hindering accurate image analysis and interpretation. *Goal:* In this study, we propose a hybrid approach to effectively segment common occlusions encountered in remote monitoring applications within PICUs. Our approach centers on creating a deep-learning pipeline for limited training data scenarios. *Methods:* First, a combination of the well-established Google DeepLabV3+ segmentation model with the transformer-based Segment Anything Model (SAM) is devised for occlusion segmentation mask proposal and refinement. We then train and validate this pipeline using a small dataset acquired from real-world PICU settings with a Microsoft Kinect camera, achieving an Intersection-over-Union (IoU) metric of 85%. *Results:* Both quantitative and qualitative analyses underscore the effectiveness of our proposed method. The proposed framework yields an overall classification performance with 92.5% accuracy, 93.8% recall, 90.3% precision, and 92.0% F1-score. Consequently, the proposed method consistently improves the predictions across all metrics, with an average of 2.75% gain in performance compared to the baseline CNN-based framework. *Conclusions:* Our proposed hybrid approach significantly enhances the segmentation of occlusions in remote patient monitoring within PICU settings. This advancement contributes to improving the quality of care for pediatric patients, addressing a critical need in clinical practice by ensuring more accurate and reliable remote monitoring.

## I. Introduction

Remote patient monitoring (RPM) [1] is a promising alternative to invasive monitoring, driven by advancements in deep learning-based computer vision (CV). CV tasks like remote photoplethysmography (R-PPG) [2], pose estimation [3], and thermal monitoring [4] provide critical, non-invasive physiological data. RPM is especially vital in pediatrics for improved access, chronic disease management, reduced hospitalizations, tailored monitoring, parental involvement, and timely intervention [5].

Downstream CV task performance relies on high-quality input data, as noise and outliers hinder accuracy. Deep learning models, especially those trained on limited data, are sensitive to irrelevant information, which must be addressed. For instance, R-PPG methods require precise regions of interest; occlusions like tubes can impair estimation accuracy [6]. Treating occlusions through localization and segmentation [7] can mitigate these issues, with semantic segmentation grouping pixels by class [8].

Deep learning's success often depends on extensive training datasets, which are scarce in PICU settings due to consent and image quality issues. Transfer learning can leverage large external datasets to improve training, using fine-tuning or zero-shot learning. However, gaps between pre-training and target medical domains can limit zero-shot performance [9], [10]. Thus, robust segmentation algorithms for environments with limited data and annotation remain crucial.

Understanding these challenges as well as being motivated by the necessity of occlusion processing in RPM applications, in this work we proposed a novel deep learning-based framework for occlusion segmentation in PICU settings. Our contributions include:

**Fig. 1.**
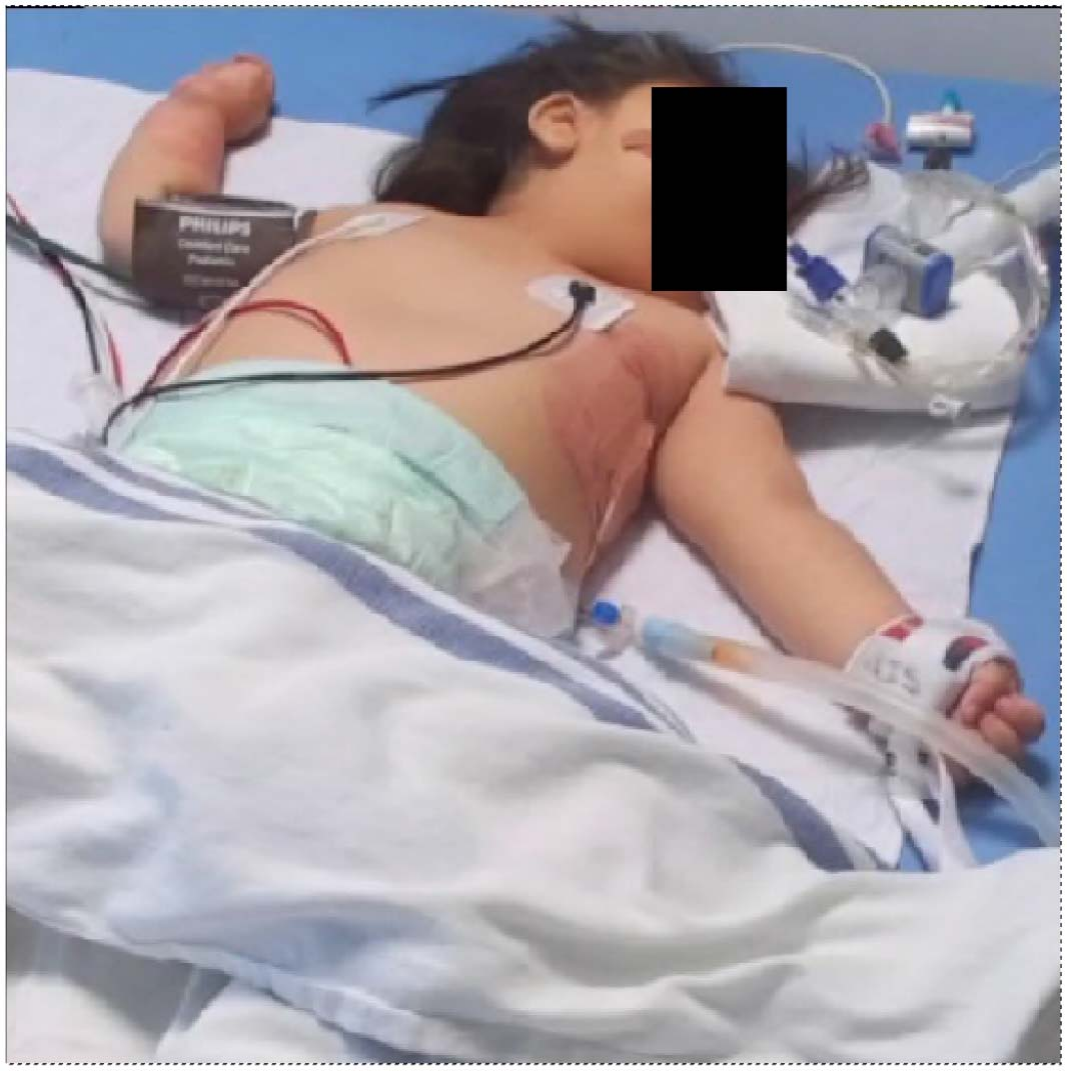
An illustrative image of a PICU patient with different occlusions in our CHU Sainte Justine's database.

**Fig. 2.**
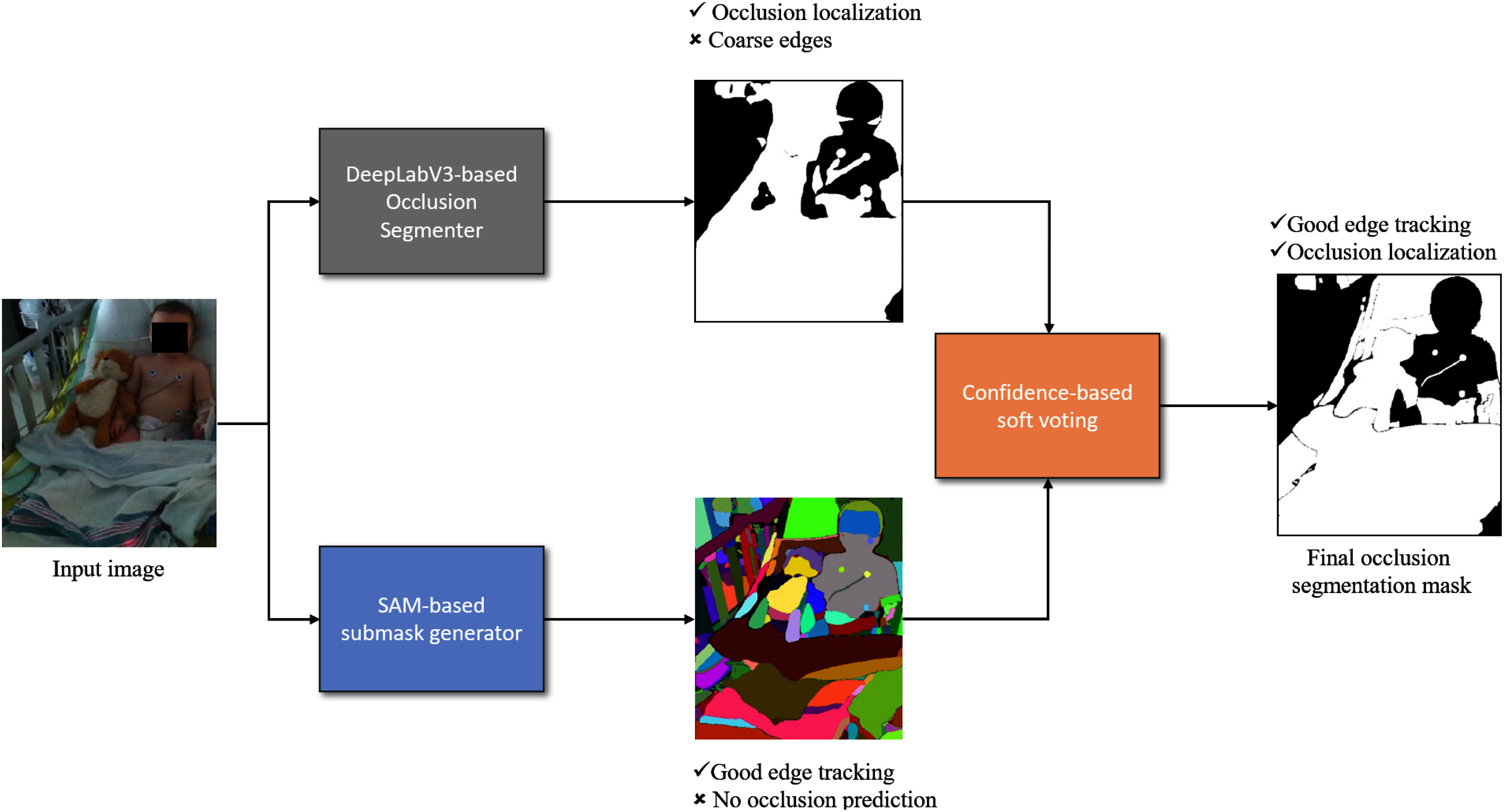
Our proposed pipeline (SOSS) for occlusion segmentation. The input image is fed to both the top branch and bottom branch simultaneously. Top branch: our DeepLabV3+-based network segments the input image and produces a semantic (occlusion) mask proposal. Bottom branch: the SAM-based generator produces a partitioning of the input image without corresponding semantic labels. Both kinds of masks are then fused using our proposed confidence-based soft voting mechanism for the final occlusion segmentation mask. This aims to add semantic information to the SAM branch while simultaneously improving the segmentation quality of the DeepLab branch.

• First, a real-world dataset of pediatric patients' images at CHU Sainte Justine Hospital (CHUSJ) with occlusions of various kinds, shapes, and sizes has been collected and annotated, which can readily be used for training and evaluating different algorithms on occlusion detection and segmentation tasks.
• Second, a convolutional neural network (CNN)-based model is trained and evaluated on the dataset, reporting plausible performance and generalization for occlusion segmentation in PICU settings where limited and unbalanced training data is a challenging problem.
• Third, a novel data-efficient fusion pipeline named SOSS (SAM-powered Occlusion Segmentation via Soft-voting) is introduced (Fig. 2). This pipeline leverages a foundation-class transformer-based image segmentation model as a means to refine the output of the preceding CNN-based occlusion segmentation model, effectively proving its capability of segmenting occlusions of various kinds in our considered clinical use case.

With this novel framework, we intend to promote the applicability of RPM for PICU deployments and other real-world uses of computer vision.

## II. Materials and Methods

In this section, we summarize related works in segmentation, and deep-learning for occlusion handling. Other relevant works about learning-based semantic segmentation are included in the supplementary documents. Additionally, we discuss our specific training methods and data pipelines.

### A. Related Works

Convolutional Neural Networks (CNNs) have been essential for semantic segmentation, utilizing convolutions for multi-scale semantic and spatial extraction. Fully Convolutional Networks (FCN) [11] and U-Net [12] introduced effective encoder-decoder structures, with U-Net excelling in preserving details via skip connections. DeepLab [13] advanced this by incorporating atrous convolutions to enhance receptive fields without excessive parameters, and DeepLabV3+ [14] further improved with ASPP and U-Net-like architectures, achieving state-of-the-art results. Despite CNNsâ efficiency, handling complex occlusions remains a challenge. Solutions like BCNet [15], ORM [7], and hierarchical models aim to model occlusion relationships and improve instance segmentation but often fall short in medical contexts with device obstructions and irregular poses. Vision transformers like Segmenter [16] and SegFormer [17] capture global context but require extensive data and may lack generalizability in data-scarce domains like pediatric ICU imaging. Our approach integrates SAM's zero-shot segmentation with DeepLabV3+ fine-tuning to enhance segmentation under complex occlusions in clinical environments, balancing pre-trained transformer strengths with tailored CNN-based precision.

### B. Methods

In this work, occlusions are defined as common objects near a PICU patient that can obstruct a direct view of the body. Background elements like walls, floors, and ceilings, as well as objects not typically on the patient's bed, are considered non-occlusions. The varying patient poses often make it challenging to locate obscured body parts under occlusions. Items such as blankets or tubes beside the patient may also be labeled as occlusions.

**Fig. 3.**
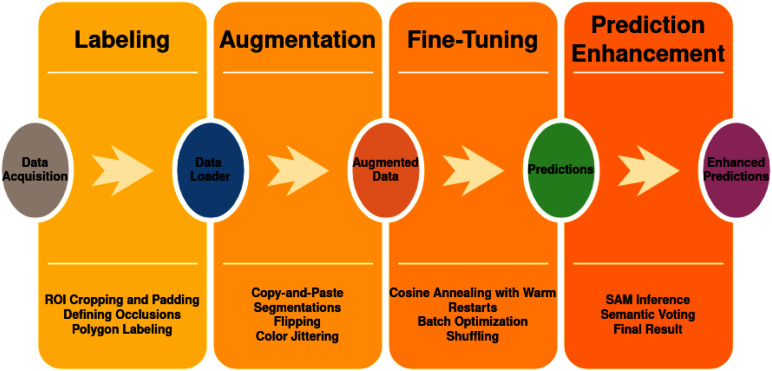
A summary of our workflow, including 4 steps: labeling, data augmentation, model fine-tuning, and prediction.

A simplified workflow is shown in Fig. 3, outlining the pipeline from data acquisition to prediction enhancement. The main stages are:

• *Data Acquisition:* Collecting images with target occlusions, processing, and storing them for annotation and segmentation.
• *Labeling:* Preprocessing (ROI cropping and padding) and annotating occluded regions using polygon labeling to ensure accurate training data.
• *Data Augmentation:* Enhancing model robustness through augmentations like flipping, color jittering, and brightness & contrast adjustments. More details are in the supplementary materials.
• *Model Fine-Tuning:* Using a DeepLabV3+ model for semantic segmentation, optimized with a cosine annealing schedule, batch optimization, and data shuffling.
• *Prediction Enhancement:* Refining initial segmentation with SAM-based image segmentation and applying semantic soft-voting for improved occlusion mask accuracy.

### C. Data Acquisition, Labeling and Data Augmentation

The study was approved by the research ethics board (REB) of CHUSJ (project number 2020-2287) and was conducted on a video database approved by the same REB (database project number 2016-1242).

In the PICU at CHUSJ, 175 high-resolution color digital photographs from consenting patients were acquired. We split the dataset into three different parts where 80% of the dataset was kept for training, 10% of the dataset was used for validation and the remaining 10% of the dataset was used for testing. Because of the ethical implications of our dataset, we cannot publicly release it; however, access to the database can be granted upon request by Dr. Philippe Jouvet.

Instance segmentation masks for occlusions were generated in the form of polygons. The different occlusion polygons were classified to better understand the most common occlusions in the PICU and how much space each occupies. Specificities on these operations are detailed in the supplementary materials.

### D. Training Semantic Segmentation Network

We trained a DeepLabV3+ network for the task of binary classification for 122 epochs on the augmented dataset. Further details are available in the supplementary materials.For the purpose of our experiments, we denote this model as our semantic segmentation model ($\mathcal {M}_\text{SEM}$).

### E. Prediction Enhancement with SAM

Although the trained $\mathcal {M}_\text{SEM}$ demonstrates potential in recognizing and localizing occlusions, its accuracy in shaping occlusions can be limited due to:

• Limited training dataset size.
• Small or thin occlusions combined with noisy, low-resolution input images.

To address these limitations, we integrated the Segment-Anything-Model (SAM), a transformer-based image segmentation model with strong zero-shot learning capabilities. SAM can be guided using prompts like bounding boxes, points, or text. However, due to the complex, overlapping nature of occlusions (e.g., cables and tubes) in PICU settings, bounding boxes were not effective. Initial experiments showed single points and text prompts were insufficient for robust segmentation.

We adopted an automatic prompting method, similar to [18], using a $40 \times 40$ point grid to prompt SAM, ensuring higher point density suited for small occlusions. We set a confidence threshold of 90%, a stability score of 85%, and a minimum segmentation area of 5 pixels to exclude irrelevant regions.

While SAM produced high-quality image segmentation with accurate boundaries, it presented limitations:

• Image segments lacked explicit semantic class associations.
• Segments might only represent parts of objects.

To leverage the strengths of $\mathcal {M}\text{SAM}$ and $\mathcal {M}\text{SEM}$, we introduced the SAM-powered Occlusion Segmentation via Soft-voting (SOSS) mechanism. Our pipeline, illustrated in Fig. 2, includes:

• A DeepLabV3+-based network generating a coarse semantic mask proposal.
• A SAM-based network providing fine-grained segmentation without semantic labels.

These outputs are combined using a confidence-based soft-voting approach to create an enhanced occlusion segmentation mask.

**Assumption 1:**

For any pair of pixel $i^\text{th}$ and $j^\text{th}$ of the same input image (I), with their respective SAM output denoted as $\mathcal {M}_\text{SAM}^{(i)}$ and $\mathcal {M}_\text{SAM}^{(j)}$, and their hidden occlusion class respectively denoted as $o^{(i)}$ and $o^{(j)}$, it is assumed that:

\begin{equation*}
\mathcal {P}\left(o^{(i)} = o^{(j)} | \mathcal {M}_\text{SAM}^{(i)} = \mathcal {M}_\text{SAM}^{(j)} \right) = 1 \tag{1}
\end{equation*}

Under this assumption, any pair of points belonging to the same segment by SAM should have the same occlusion class.

This implies that SAM segments can be regarded as the finest granularity for semantic segmentation. In fact, this assumption is supported by the observations made by the original paper [18] for object proposal tasks, instance segmentation tasks, as well as SAM's potential to carry semantic information in the latent embedding space without explicit semantic supervision. This assumption is very powerful since it allows us to effectively constrain the semantic relationship of neighboring pixels and integrate SAM into more complicated tasks in a flexible fashion.

Based on this assumption, we then proposed our fusion algorithm (SAM-powered Occlusion Segmentation via Soft-voting, or SOSS for short) as follows:

Algorithm 1: **SAM-Powered Occlusion Segmentation via Soft-Voting (SOSS).**
PREDICT $(\mathbf {\mathcal {M}_\text{SEM}}$ $ \mathbf {\mathcal {M}_\text{SAM}}$ $\mathbf {I})$

$ \mathbf {j} = (1,1)$

$ \mathbf {P_{SAM}} \gets \mathbf {\mathcal {M}_\text{SAM}}(I)$

$ \mathbf {P_{SEM}} \gets \mathbf {\mathcal {M}_\text{SEM}}(I)$

$ \forall$ $ \mathbf {S} \in \mathbf {P_{SAM}}:$

$ P_{mod} \gets P_{SEM}S$

$ C \gets \arg \max (P_{mod}j)$

$ \mathbf {P_{FINAL}} \gets C\mathbf {S}+\mathbf {P_{FINAL}}$

**return** $\mathbf {P_{FINAL}}$

Using these settings, any image ($I$) of size $H\times W$ can be used by our $\mathcal {M}_\text{SAM}$ to infer a binary prediction matrix ($P_{SAM}$). Given a total number of $O$ separate objects detected by $\mathcal {M}_\text{SAM}$, $P_{SAM}$ will be of $(H,W,O)$ dimensions. Therefore, inside $P_{SAM}$, there are $O$ binary 2-dimensional matrices ($B$), each representing a “patch” (equivalently, “superpixel” or segment proposal) which corresponds to the filled silhouette of different objects in the image. These $O$ matrices are believed to have better edges than the semantic predictions of $P_{SEM}$ after applying any given threshold. In order to leverage the classification aspect of our $\mathcal {M}_\text{SEM}$ and the sharp segmentation masks of our $\mathcal {M}_\text{SAM}$, we used a soft-voting algorithm. This algorithm attributes a class $C$ from $N$ different classes to each matrix $B$ in $P_{SAM}$. In order to calculate which class to attribute, we mask $P_{SEM}$ along its $H$ and $W$ dimensions using every matrix $B$ in $P_{SAM}$. The resulting masked prediction ($P_{mod}$) is used to determine the class C by locating the index of the maximum semantic confidence value. To do so, we sum $P_{mod}$ along its $N$ dimension, resulting in a uni-dimensional vector of length $N$. The index of the maximum value in this vector corresponds to the final occlusion class $C$.

The algorithm's intuition is that SAM's accurate segmentation allows querying all pixels in each segment for their occlusion classes from $\mathcal {M}\text{SEM}$. The class with the highest votes is selected as the final segment prediction. To mitigate $\mathcal {M}\text{SEM}$’s overconfidence due to limited training data, a soft-voting mechanism is used instead of hard voting, ensuring conservative polling. This fusion approach addresses SAM's limitations by merging segments where the majority of pixels agree on a class, creating unified segments with semantic labels. It also refines $\mathcal {M}_\text{SEM}$’s outputs by forming semantic segments with more precise boundaries.

## III. Results

A variety of tools have been used to implement our proposed pipeline. To annotate the dataset we used the LabelStudio [19] toolset. The training of our $\mathcal {M}_\text{SEM}$ network was done using Pytorch[20]. The data was also augmented using the Albumentations [21] library, besides the CopyPaste augmentation that was made to fit as another transform in the same pipeline. We also leveraged OpenCV[22] and Numpy [23] for image manipulation. To evaluate the pipeline, a held-out test set consisting of 10% of the total images was used without any augmentations.

To alleviate the effect of class imbalance, which can be strong in our dataset, we included the following metrics for performance comparison:

• Accuracy: $Acc=\frac{TP+TN}{TP+TN+FP+FN}$
• Precision: $Prec=\frac{TP}{TP+FP}$
• Recall: $Rec=\frac{TP}{TP+FN}$
• F1 score: $F_{1}=(\frac{0.5}{Prec}+\frac{0.5}{Rec})^{-1}$
• Intersection over Union (IoU): $IoU= \frac{|True \cap Predicted|}{|True \cup Predicted|}$
• Area Under the receiver operating characteristic Curve (AUC)

where F and T stand for False and True; P and N stand for Positive and Negative, respectively. Furthermore, TN and TP stand for true negative and true positive respectively, and are the number of negative and positive cases correctly classified. FP and FN represent false positives and false negatives and the number of incorrectly predicted positive and negative cases. These metrics are per class; we report them for the occlusion class (class 1) in the following discussion. In this work, a true positive is defined as any pixel within the ROI that is correctly classified as an occlusion (class 1) based on the binary ground truth mask. The ground truth occlusions are represented as binary masks encompassing annotated polygon areas, which may include portions of the body or other structures due to the nature of occlusions in clinical settings. Therefore, a detected occlusion is counted as a true positive if it overlaps with any part of the ground truth occlusion mask, even if the outline does not perfectly match the occlusion's exact shape. This approach ensures that minor variations in the segmentation boundaries do not penalize the model's performance excessively.

Specifically, Table I shows the occlusion segmentation performance of our $\mathcal {M}_\text{SEM}$, and then those attained by the whole proposed SOSS pipeline. To ensure statistical rigor, we conducted the training and testing over random split and reported the average performance. The reported results were obtained using a three-part split of the dataset, where 80% of the images were used for training, 10% for validation, and the remaining 10% for testing. We can see that the proposed method consistently improves the predictions across all metrics, with an average of 2.75% gain in performance. Note that our performance evaluation considered all occlusions in the image, including those in both the foreground and the patient's side regions. Focusing solely on foreground occlusions would likely have resulted in a more significant performance gain because these regions are typically less ambiguous and easier to segment. However, our current analysis does not exclude patient-side occlusions, as we aimed to comprehensively evaluate the segmentation model across different contexts. Overall, this increase clearly demonstrates the effectiveness of our proposed pipeline in the intended task of occlusion segmentation.

**TABLE I:** Performance of CHU-SJ artifact segmentation task

Metrics	DLV3+	SOSS (ours)
$Accuracy \uparrow$	89.1	**92.5**
$F1 \uparrow$	90.0	**92.0**
$Precision\uparrow$	88.1	**90.3**
$Recall \uparrow$	92.0	**93.8**
$IoU \uparrow$	81.8	**85.2**
$AUC \uparrow$	88.9	**92.6**

Bold denotes the best values.

## IV. Discussion

Figs. 5 and 6 illustrate test set samples with corresponding predictions at various pipeline stages to analyze the improvements brought by the proposed method.

**Fig. 4.**
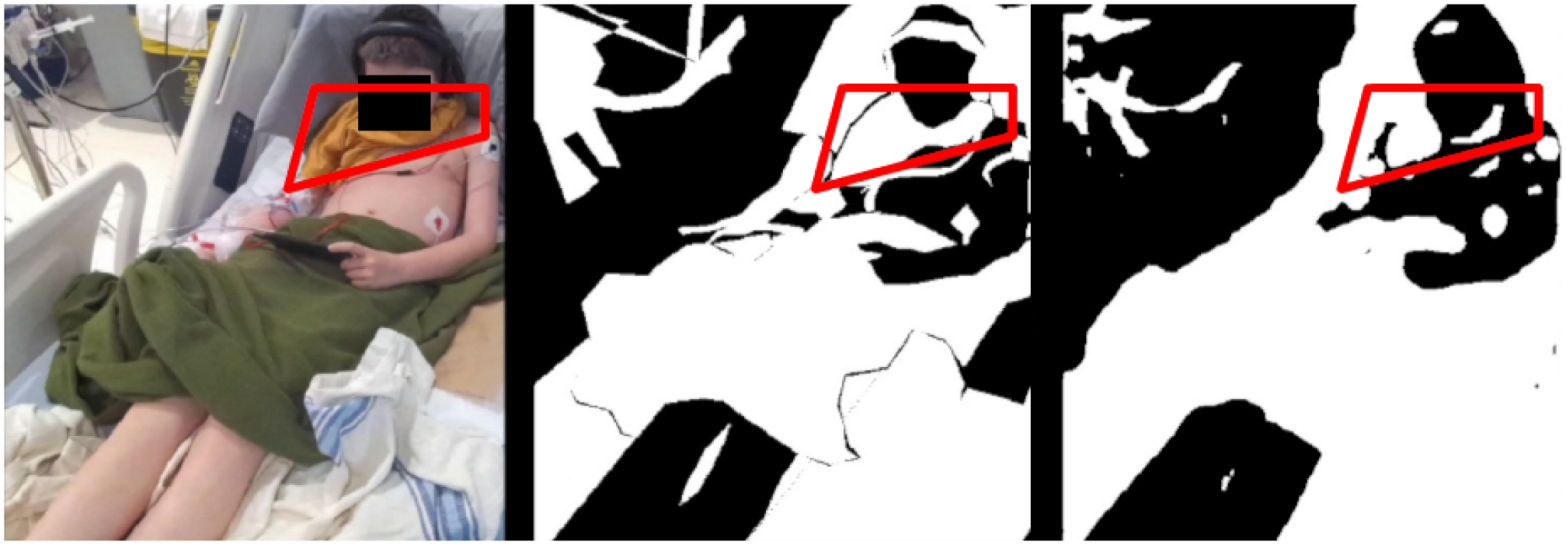
An example of $\mathcal {M}_\text{SEM}$’s prediction. (Left) Input image. (Middle) Occlusion segmentation annotation. (Right) Occlusion segmentation mask by $\mathcal {M}_\text{SEM}$. The scarf (highlighted in the red box) is roughly localized and identified as occlusion. However, the predicted mask shape of the scarf is inaccurate.

**Fig. 5.**
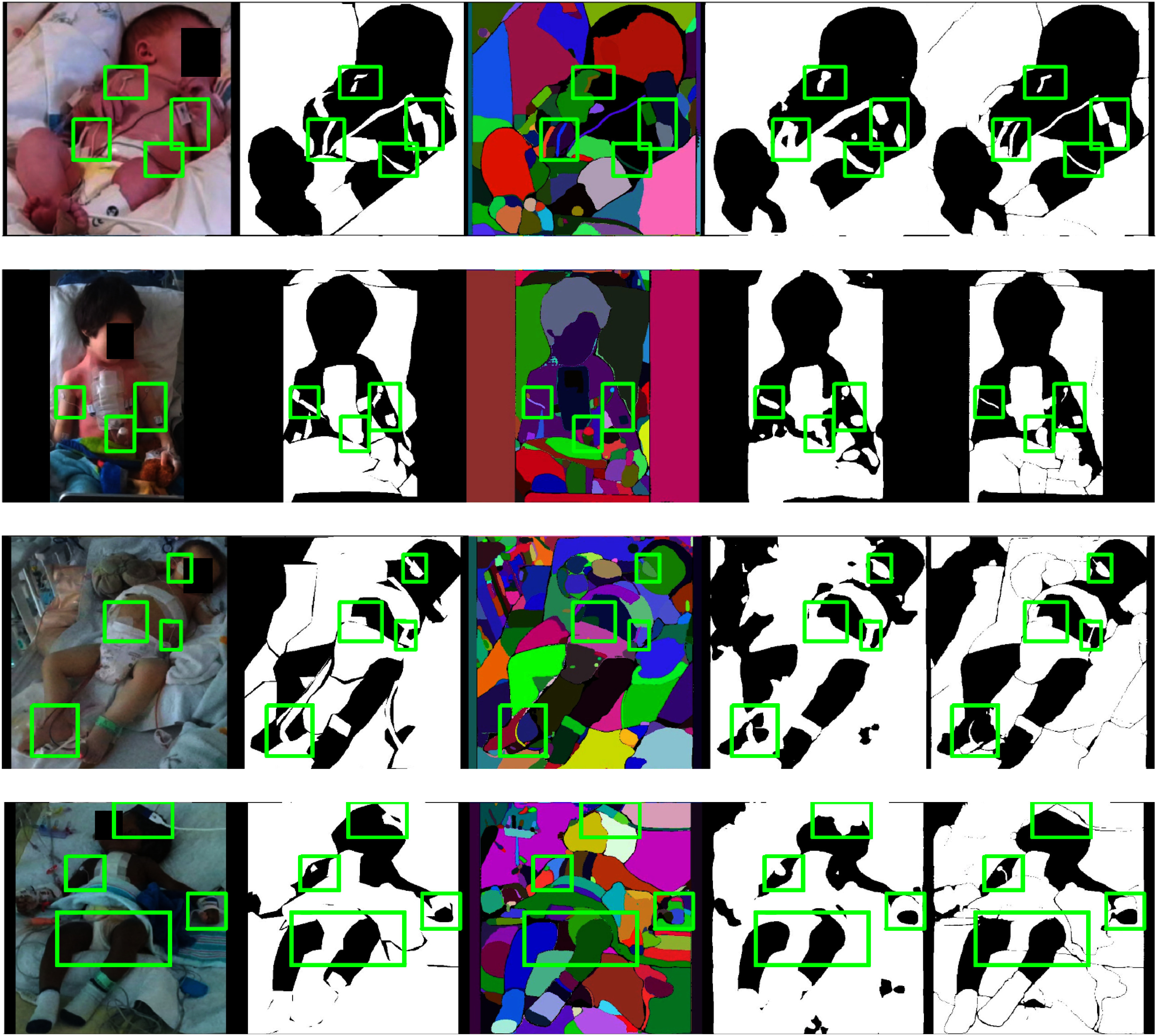
Improved predictions on the held-out test data. From left to right: input images ($1^\text{st}$ column), ground truths ($2^\text{nd}$ column), SAM masks ($3^\text{rd}$ column), predictions from $\mathcal {M}_\text{SEM}$ ($4^\text{th}$ column), and final binary occlusion segmentation mask ($5^\text{th}$ column). In the $2^\text{nd}, 4^\text{th}$ and $5^\text{th}$ columns, white regions are classified as occlusions while black ones are classified as non-occlusions. In the $3^\text{rd}$ column, different colors simply indicate different image segments without associated occlusion class, which is the main shortcoming of SAM in our task. Green boxes highlight occlusion easily visible segmentation improvements.

**Fig. 6.**
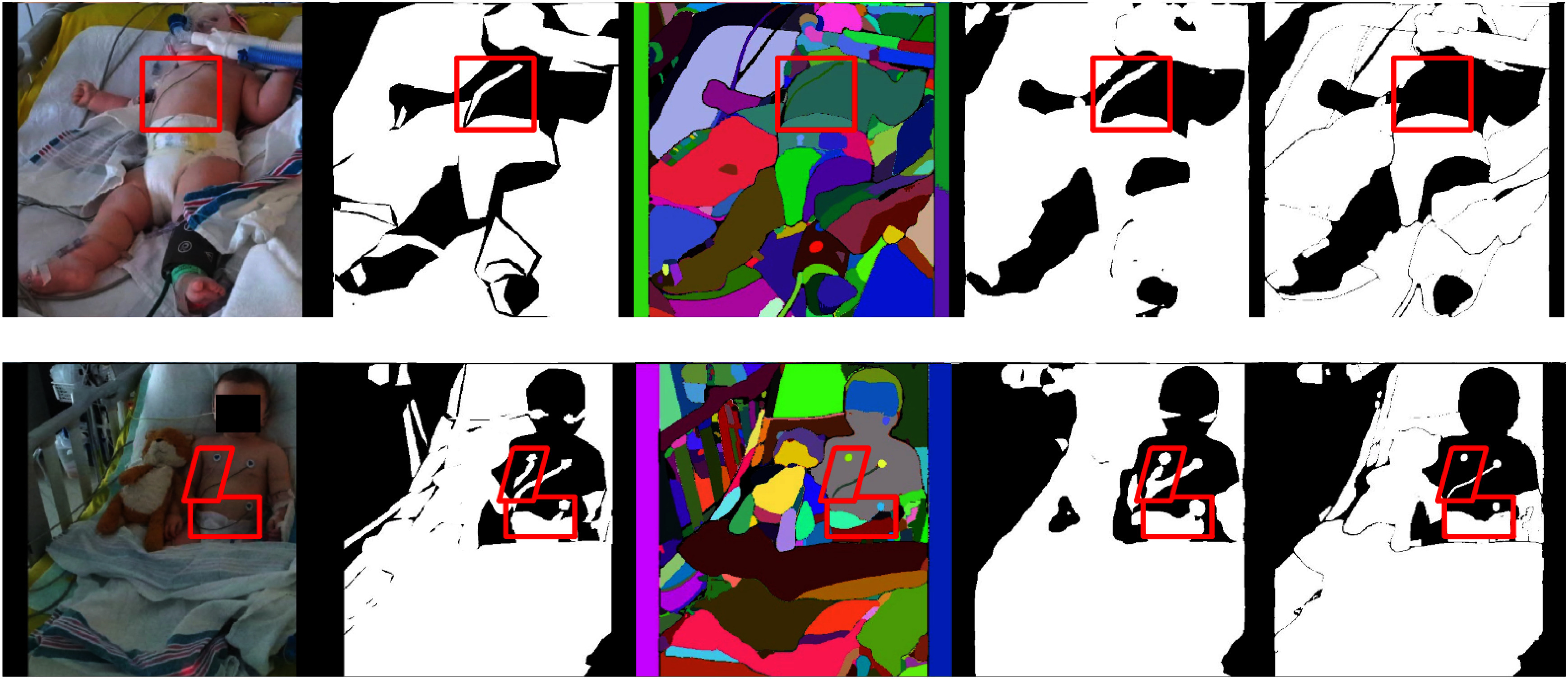
Failure examples of SOSS. From left to right: input images ($1^\text{st}$ column), ground truths ($2^\text{nd}$ column), SAM masks ($3^\text{rd}$ column), predictions from $\mathcal {M}_\text{SEM}$ ($4^\text{th}$ column), and final binary occlusion segmentation mask ($5^\text{th}$ column). In the $2^\text{nd}, 4^\text{th}$ and $5^\text{th}$ columns, white regions are classified as occlusions while black ones are classified as non-occlusions. In the $3^\text{rd}$ column, different colors simply indicate different image segments without associated occlusion class, which is the main shortcoming of SAM in our task. Red boxes highlight occlusion segmentation failures.

By comparing the right-most columns with the input images (*1st* column) and the occlusion mask annotations (*2nd* column), the quality of segmentation masks at each step can be assessed. For example, in the second row of Fig. 5, a pediatric patient in the PICU shows a fine cable attached to the right arm. While SAM (*3rd* column) provides precise segmentation, it lacks semantic labels. In contrast, $\mathcal {M}_\text{SEM}$ (*4th* column) assigns occlusion classes but may produce coarser shapes. The SOSS predictions (*5th* column) accurately capture both the label and shape.

Visual inspection reveals that $\mathcal {M}_\text{SEM}$ effectively localizes occlusions despite limited training data, handling varied illumination, patient demographics, and dense occlusions with stable boundaries, albeit with occasional over-smoothing and over-representation of thin objects.

SAM's predictions, though accurate in segment shape, lack semantic labeling, leading to ambiguity. The SOSS method improves segmentation quality by:

• Preserving fine details of thin or small objects and aligning segments closely with the ground truth.
• Recovering missing details from $\mathcal {M}_\text{SEM}$ and maintaining small objects like electrodes and patches.

Failure cases of SOSS, shown in Fig. 6, highlight challenges with thin objects like cables. The pipeline's reliance on SAM can lead to missed detections if assumptions about SAM's completeness are unmet.

In summary, combining DeepLabV3+ and SAM yields a 2.75% performance gain on average and enhances occlusion segmentation in clinical settings, even with limited data. However, limitations include sensitivity to small occlusions and increased computational complexity from SAM's detailed masks, affecting inference speed. Despite these challenges, integrating SAM with CNN models through a soft-voting mechanism improves overall robustness.

## V. Conclusion

In this paper, we proposed a pipeline for efficiently segmenting occlusions in a clinical setting with little data by leveraging pre-trained semantic segmentation models, data augmentation, and mature promptable segmentation models like SAM. Our findings suggest that efficient segmentation of occlusions in a PICU setting is a task that can be accomplished with limited data and the help of strong zero-shot segmentation models.

On the other hand, there are certain shortcomings associated with our methodology, mostly associated with SAM:

• *Slow inference:* The main bottleneck in our pipeline is SAM, which takes significantly longer time for inference compared to $\mathcal {M}_\text{SEM}$.
• *Prompting resolution:* Masks generated by SAM can be quite sensitive to the chosen prompting resolution. SAM can skip objects because of their small or thin size, as in the case of cables that perhaps fall between prompting points in the image. Generally, more prompted points require higher computing resources. This is however limited by the computational power of the existing hospital's dedicated server; in other words, sufficiently fine-grained point prompting might not be attainable.
• *Overconfidence in SAM:* Our proposed pipeline is based on the assumption of SAM accuracy. Even though SAM's predictions are very accurate in general, there are certain cases where SAM is unaware of very small, irregular objects.

Therefore, our future research can be extended in the following directions:

• *Utilizing SAM in a more efficient way:* For example, we can optimize the prompting resolution to balance between accuracy and inference speed. Another possible improvement is to use faster implementation of SAM, for example with a recently proposed architecture in [24].
• *Misclassification analysis:* We plan to conduct a comprehensive error analysis to identify specific occlusion types that are challenging for the model to recognize. This will involve comparing multi-class manual labels with the model's predictions to identify common misclassifications and refine our framework, improving accuracy and robustness.
• *Leveraging multi-modality:* Additional synchronized modalities such as depth images and thermal images captured by the hospital acquisition system can be provided for our dataset. SAM is also reported to work with depth modality [25] and thus can exploit rich geometric information from depth images aside from textural information given by RGB images, which might further boost the segmentation performance.
